# Microencapsulation of Purple Mashua Extracts Using Andean Tuber Starches Modified by Octenyl Succinic Anhydride

**DOI:** 10.1155/2022/8133970

**Published:** 2022-01-25

**Authors:** Frank Fluker Velásquez-Barreto, Carmen Eloisa Velezmoro Sánchez

**Affiliations:** ^1^Programa Doctoral de Ciencia de Alimentos, Escuela de Posgrado, Universidad Nacional Agraria La Molina, Av. La Molina s/n, La Molina, Lima 15024, Peru; ^2^Escuela Profesional de Ingeniería Agroindustrial, Facultad de Ciencias Agrarias, Universidad Nacional Autónoma de Chota, Colpa Huacaríz, Chota, Cajamarca 06120, Peru

## Abstract

This work is aimed at optimising the spray drying conditions of the phenolic extracts of purple mashua microencapsulated with octenyl succinic anhydride (OSA) Andean tuber starches. Purple mashua extracts were obtained and spray dried using native and OSA starches of yellow oca, pink oca, and yellow olluco (140°C, 4% starch). The powders obtained were analysed by encapsulation efficiency of anthocyanin (EE), total phenol content, and antioxidant capacity to select the best starch for optimisation purposes. OSA pink oca starch was selected because the obtained powder presented the highest EE. The spray drying conditions optimised were obtained using a central composite rotatable design (CCRD) and response surface methodology. The encapsulant proportion of OSA pink oca starch (2-12%) and the inlet drying temperature (IDT, 120-160°C) were used as factors of the design. The optimised spray drying condition was 160°C IDT and 2% encapsulant; this condition maximised the EE, total phenol content, antioxidant capacity, and solubility and minimised the water activity and hygroscopicity of the powder. The OSA pink oca starch could be used as an encapsulating agent of phenolic extracts because it can produce powder with high antioxidant capacity and high EE.

## 1. Introduction

Unconventional tubers such as mashua, oca, and olluco are cultivated in the Andean region of South America and have been used since Inca times. Oca and olluco are consumed in raw or cooked form in culinary highlights. Starch is the major constituent of these tubers. Certain studies of native oca and olluco starches indicated that they have a good paste forming property, low gelatinisation temperature, and high elasticity of gels [1–3]. Nevertheless, native starches require certain types of physical or chemical modification to enhance their physicochemical and functional properties [4, 5].

Different types of chemical processes such as acetylation, succinylation, and oxidation are used for modifying starches. One of the commonly used modification methods is one using octenyl succinic anhydride (OSA), which involves the inclusion of OSA groups in the starch chains, subsequently producing esters that have hydrophilic and hydrophobic properties. Starches with these properties can be used for various processes including gel formation, encapsulation, and emulsification. Because of their aroma preservation properties and protection of hydrophilic and hydrophobic bioactive compounds, starches esterified by OSA are used to microencapsulate certain extracts such as eggplant [6], tomato concentrate [7], and sweet palm [8].

Mashua is an Andean tuber distributed in South American highlands, and its average proximal chemical composition is 84.5% humidity, 15.5% total solids, 7.7% protein, 1% fat, 0.7% fibre, 4.8% ash, and 85.8% carbohydrates, expressed on the dry basis of the product [9]. The bioactive compounds of different varieties of mashua tubers such as phenolic antioxidants, glucosinolates, anthocyanins, carotenes, and other pigments possess certain properties that could fight against degenerative diseases such as cancer, cardiovascular, and diabetes [10, 11].

Spray drying is an alternative method for producing powder-based products with high antioxidant content. This method is extensively used in the microencapsulation of food ingredients that are susceptible to deterioration by external agents. In this method, an active agent (solid particles, liquid droplets, or gaseous compounds) is trapped in a polymer matrix to protect it from adverse conditions [12]. The microencapsulation process has been used in the food industry to protect sensitive food ingredients during storage, to mask or preserve aroma and flavours, to protect food from nutritional losses, or even to add nutritional materials in the food after processing [13]. The encapsulation of plants extracts is usually obtained using gums, maltodextrins, and certain modified starches as wall materials [7, 14, 15].

To prevent the degradation of food items and nonfood items, the existing trend is to extract the bioactive compounds present in them and maintain their characteristics using certain methods. Different factors, such as temperature and light, can affect their stability, and then, the microencapsulation process can be used to prevent it. Researches are looking for novel wall materials to microencapsulate bioactive compounds of food extracts with bioactive compounds. Native starches of Andean tubers can be used to, further, these starches; however, they need to be modified by certain methods to improve their functional properties. Therefore, this study is aimed at optimising microencapsulation by spray drying bioactive compounds of purple mashua extracts (PME) using Andean tuber starches modified with OSA.

## 2. Materials and Methods

### 2.1. Materials

Purple mashua tubers (*Tropaeolum tuberosum* Ruiz and Pavón) were obtained by harvesting from Tinquerccasa locality, 3600 m of altitude (Paucará district-Huancavelica-Perú). OSA-modified starches of yellow oca (*Oxalis tuberosa*) with degree substitution (DS) of 0.014, pink oca (*Oxalis tuberosa*) with DS 0.016, and yellow olluco (*Ullucus tuberosum*) with DS 0.015 were used to encapsulation agents. These modified starches were obtained in our previous work [3, 16]. Chemical reagents were purchased from Merck.

### 2.2. Microencapsulation of PME

#### 2.2.1. Preparation of PME

To obtain the PME, 3 kg of clean purple mashua tubers without any physical damage or defect was cut into cubes of sides that are 2 mm and placed in a dark container. Then, 4.5 L of distilled water was added, and leaching extraction was performed by soaking the cubes of tubers for 24 h at 4°C. Subsequently, the extracts were filtered in gauze and organza cloth and then centrifuged at 4000 rpm for 10 min to remove residues and suspended materials. The obtained supernatant (mashua extracts) was stored at −18°C in polyethylene terephthalate bottles covered with Al foil. The total solid content of the extracts was 2 Brix. Extracts exhibited an anthocyanin content of 38.05 mg of cyanidin-3-glucoside/L, antioxidant capacity in 2,2′-diphenyl-1-picrylhydrazyl (DPPH) of 100.29 *μ*mol Trolox/100 mL, and total phenol content of 80.7 mg gallic acid/100 mL.

#### 2.2.2. Selection of OSA Starch to Microencapsulate the PME

The spray drying process was used for selecting the most appropriate modified starch for microencapsulation. Spray drying of PME was performed using native and OSA-modified starches as the encapsulating agents. Starch was added to 200 mL of extract in a concentration of 4% (*w*/*w*) (Table 1). This concentration was selected in preliminary experiments to produce powder without excessive powder stickiness on the chamber wall [17]. The suspensions were homogenized at 4000 rpm in a homogenizer (Ultraturrax T18, IKA, Germany) and atomised in a mini spray drier (B-290, Buchi Labortechnik AG, Flawil, Switzerland) at 140°C. The process conditions were 10% of feed (3 mL/min), 35 m^3^/h aspiration (90%), and 40 mm of air flow (473 L/h). Furthermore, the microcapsules were placed in glass containers with a lid and protected from light by covering them with Al foil. Then, the containers were stored at room temperature, and the encapsulation efficiency of anthocyanins (EE), total phenol content, and antioxidant capacity of the powder obtained were analysed. The OSA-modified pink oca starch was selected because it presented the highest EE.

#### 2.2.3. Optimisation of the Microencapsulation of PME

The OSA-modified pink oca starch was used as the encapsulating agent to optimise the spray drying process conditions, because of its high EE (Section 2.2.2). The central composite rotatable design (CCRD) and the response surface methodology were used to analyse and evaluate the effects of the inlet drying temperature (IDT) and encapsulant proportion (OSA-modified pink oca starch) on the EE, total phenol content, antioxidant capacity, water activity (*a*_*w*_), hygroscopicity, and solubility of the spray-dried PME. The CCRD was a 2*k* factorial design where *k* is equal to two factors with 2*k* axial points (of value 1.41) and four additional centre points. The IDT and the encapsulant proportion were selected using a preliminary study and the methodology proposed by Tonon et al. [17] to avoid the stickiness of the powder in the chamber. In the CCRD, the encapsulant proportion varied from 2% to 12%, while the IDT varied from 120°C to 160°C (Table 2). In addition, the total runs were 12. The spray drying of PME was performed as per the procedure described in Section 2.2.2.

The results were adjusted to a second-order polynomial model according to the following equation:

(1) $$\begin{array}{c} Y=\beta_{0}+\beta_{1}X_{1}+\beta_{2}X_{2}+\beta_{11}X_{11}+\beta_{22}X_{22}+\beta_{12}X_{22}, \end{array}$$

where *Y* is the response, *β*_1_ and *β*_2_ are linear coefficients, *β*_12_ is a cross-product coefficient, and *β*_11_ and *β*_22_ are the quadratic coefficients for variable *Xi* (encapsulant proportion and IDT).

The results were analysed, and a mathematical model was determined for each response as a function of the IDT and encapsulant proportion. The fitted models were used to determine the optimum conditions of the spray drying process using the multiple response analysis of the desirability function [18].

### 2.3. Characterization of PME Powder

#### 2.3.1. Anthocyanin Content

The differential pH method was used for the quantification of anthocyanin [19, 20].

#### 2.3.2. EE

The EE was determined on the basis of the total anthocyanin content (TAC) and surface anthocyanin content (SAC) of microcapsules as per the method proposed by Aguilar-Tuesta et al. [20]. The EE was calculated using

(2) $$\begin{array}{c} \text{EE }\left(\%\right)=\frac{\left(\text{TAC}-\text{SAC}\right)}{\text{TAC}}\times 100. \end{array}$$

#### 2.3.3. Antioxidant Capacity

The antioxidant activity was determined using a free radical capture method of DPPH [21, 22]. The samples were analysed in duplicate, and results were represented as *μ*mol Trolox Equivalent (TE) per gram of powder.

#### 2.3.4. Total Phenol Content

The method reported by Heimler et al. [23] was used to analyse the total phenol content, which is based on the Folin-Ciocalteu method. The total phenol content was determined using a calibration curve of 20–500 *μ*g of gallic acid/mL, and it was expressed in mg of equivalent gallic acid per gram of powder.

#### 2.3.5. Water Activity

A water activity meter (Aqualab series 4TE, Meter Group, USA) was used at 25°C ± 1°C to determine *a*_*w*_ of powder; the values were expressed from zero to one.

#### 2.3.6. Hygroscopicity

Hygroscopicity is calculated as the mass of moisture (g) absorbed by g of powder as equilibrium with the environment. Hygroscopicity was determined at 25°C ± 1°C using 1 g of atomised sample, which was placed on a plate in a desiccator containing a saturated solution of NaCl (76% relative humidity). The sample was weighed after 10 days (at this time, the powder did not show any weight change), and the hygroscopicity was expressed in grams of water per gram of powder × 100 (% dry base) [24].

#### 2.3.7. Solubility

The solubility of the powder was determined using the procedure described by Pires and Pena [24] The solubility was determined in percentage based on the difference of dry residue weight and dry base weight of the sample.

### 2.4. Statistical Analysis

The experimental data of the selection of OSA-modified starches by spray drying were analysed by performing Turkey's test with SPSS 16.0. For the optimisation purpose, mathematical models were fitted to experimental data using multiple regression analysis with the help of Design Expert (version 8.0.2, Stat-Ease Inc., USA). A multiple response analysis (desirability function range 0–1) was performed to determine the optimum drying conditions using the same software.

## 3. Results and Discussion

### 3.1. Selection of OSA Starches

Table 1 lists the EE, total phenol content, and antioxidant capacity of PME that were atomised using different OSA-modified and native starches of Andean tubers. Powders obtained with OSA-modified starches demonstrated the highest EE of monomeric anthocyanin compared to the native starches. In general, the native starches of Andean tubers used to encapsulate the PME show a variation in the total phenol content and antioxidant capacity after spray drying. The powder with OSA-modified pink oca starch had the highest EE and antioxidant capacity, followed by OSA-modified yellow olluco and yellow oca starches. This difference in contents between native and OSA-modified starches is attributed to changes in the structure of native starches after the inclusion of the OSA group. Moreover, the OSA-modified pink oca starch showed the maximum OSA group content, thus resulting in the highest EE and antioxidant capacity of the PME powder. This behaviour could be because the anthocyanins enter the modified granules to bind with certain amylose and amylopectin chains with OSA groups [3, 25], thus in high retention of anthocyanins and other phenolic compounds present in the PME.

### 3.2. Effect of Independent Variables on the Responses of the Powder

#### 3.2.1. EE

**Figure 1:**
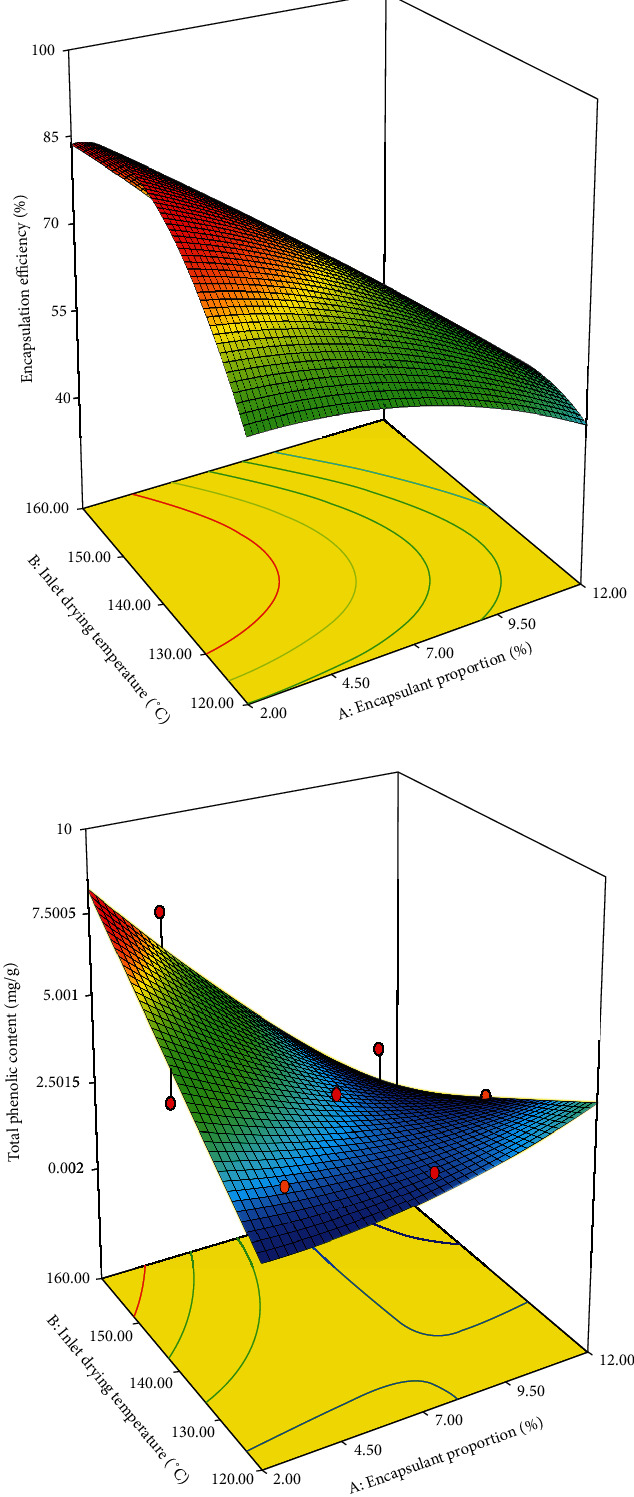
Effect of IDT and encapsulant proportion on EE (a) and total phenol content (b) of PME powder.

Figure 1(a) shows the effect of independent variables on the EE of the powder of PME. The EE of anthocyanins increased at a low encapsulant proportion (OSA-modified pink oca starch) and high IDT (Figure 1(a)). The range of EE was 35.29%–84.31% (Table 2). The maximum EE (84.31%) was observed for the powder spray dried with 2% encapsulant at an IDT of 140°C, followed by that with 3.5% encapsulant at 154°C IDT. The high EE at a high IDT and a low encapsulant proportion can be associated with the layer formation on the surface, which creates a crust (gelatinised starch), thus limiting the migration of anthocyanins from the crust of the powder to the surface [24, 26]. Similarly, the high EE of OSA-modified pink oca starch confirms the capability of OSA-modified starch to retain the high concentration of anthocyanins. The results of EE of our study were similar to the range reported by da Rosa et al. [27] for blueberry extract powder and Lao and Giusti [28] for purple corn extract powder.  Table 3 shows that the EE of anthocyanins in the powder was significantly affected (*p* < 0.05) by the encapsulant proportion and not significantly affected (*p* > 0.05) by IDT.

#### 3.2.2. Total Phenol Content

Figure 1(b) shows the relation between the independent variables and total phenol content of the powder of PME. PME spray dried with a low proportion of the encapsulant at a high IDT produced a powder with high total phenol content; this behaviour is similar to the behaviour seen for the EE. The atomised powder of PME spray dried with 3.5% encapsulant at 154°C produced the highest total phenol content. This demonstrates that the polyphenols are protected at high IDT because of the gelatinisation of the OSA-modified pink oca starch. OSA-modified starches increased the size of starches and their swelling capacity [3], which could have contributed to their increased total phenolic compound content. However, a low total phenol content of the powder was observed with a low encapsulant proportion at a low IDT (Table 2). The total phenol content was reported to range 1.96–7.84 mg/g of the atomised powder of PME (Table 2). Similar total phenol content values were reported by Zhang et al. [29] for cranberry juice powder and Saikia et al. [30] for khasi mandarin orange, watermelon, carambola, and pineapple extract powder. This shows that the OSA-modified pink oca starch could be used as an encapsulating agent of PME, because the powder shows high phenolic compounds.  Table 3 clearly shows that the effect of encapsulant proportion was significant (*p* < 0.05), while that of IDT was not significant (*p* > 0.05) on the total phenol content.

#### 3.2.3. Antioxidant Capacity

**Figure 2:**
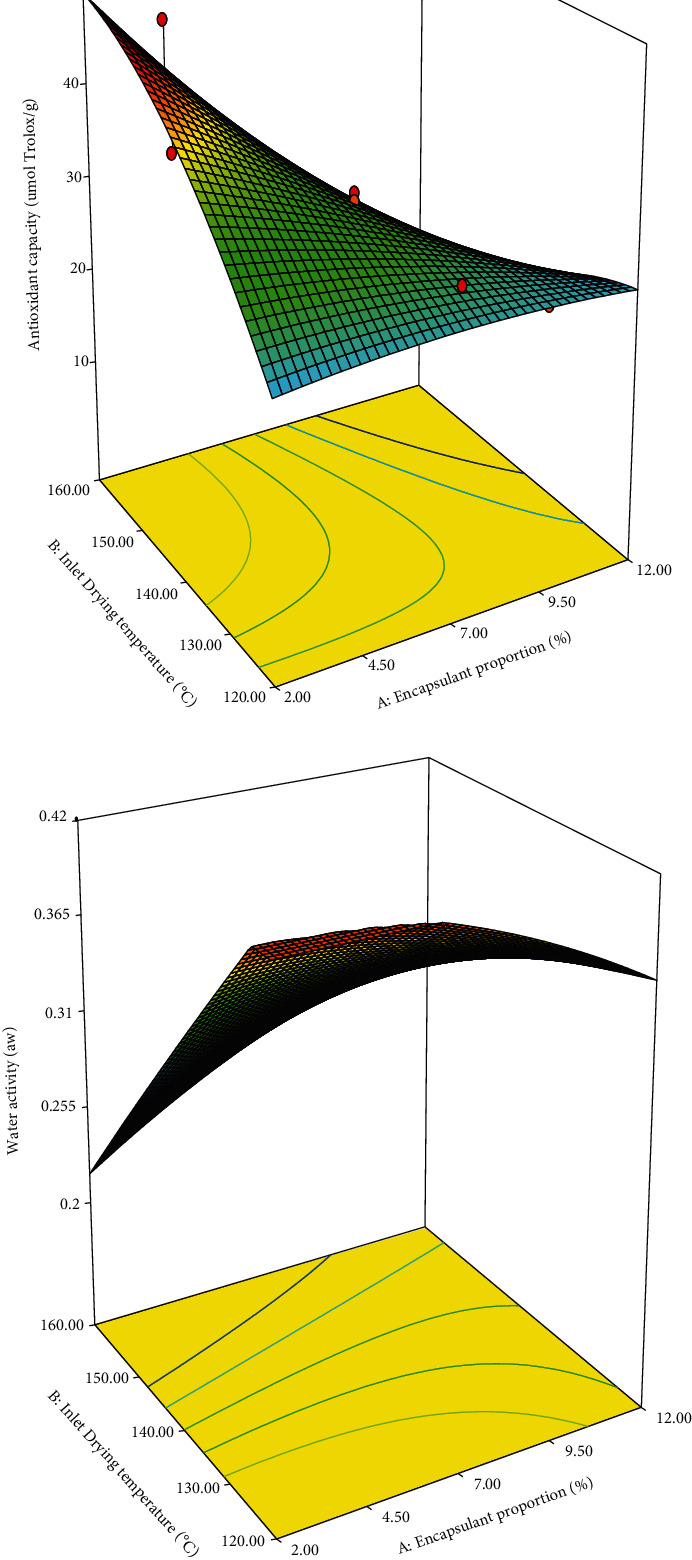
Effect of IDT and encapsulant proportion on antioxidant capacity (a) and *a*_*w*_ (b) of PME powder.

Figure 2(a) shows the effect of the independent variables on the antioxidant capacity of the PME powder. The antioxidant capacity of the PME powder increased at a high IDT and a low encapsulant proportion. Similar behaviours were reported by EE and total phenol content (Figures 1(a) and 1(b)). Anthocyanins and total phenol content positively correlate with the antioxidant capacity [31]. Therefore, high antioxidant capacity in the powder of PME could be attributed to high anthocyanins and total phenol content in the powder. Moreover, IDT lower than 140°C and encapsulant proportions greater than 3.5% reduced the antioxidant capacity perhaps because of the dilution effect (Table 2). The antioxidant capacity of the powder ranged 18.07–47.83 *μ*mol Trolox/g, which is similar to the range reported by Carvalho et al. [21] for jussara extract powder and Zhang et al. [29] for cranberry juice powder.  Table 3 shows that the effects of encapsulant proportion and the interactions of the variables are significant (*p* < 0.05) while that of IDT was not significant (*p* > 0.05) on antioxidant capacity.

#### 3.2.4. *a*_*w*_

Figure 2(b) shows that a high IDT and a low encapsulant proportion reduced *a*_*w*_ of the powder, while a high encapsulant proportion and a low IDT increased *a*_*w*_ of the powder. This behaviour is similar to previously reported trends; that is, a high IDT reduces *a*_*w*_ of the powder and an increased encapsulant proportion allows a high amount of water molecules in the powder [17, 24]. Table 2 shows that the values of *a*_*w*_ of the powder of PME spray dried with encapsulating agents ranged 0.27–0.44. The different results of *a*_*w*_ were reported by da Rosa et al. [27] for blueberry extract powder and Carvalho et al. [21] for jussara extract powder. The difference between these results and our results is the type of carrier agent used, spray drying conditions, and the extract composition. All the *a*_*w*_ values of the powder were <0.6, which indicates that the atomisation treatment at all working conditions of the experiments led to the microbiological stability of the powder [24].  Table 3 shows that the IDT has a significant effect on *a*_*w*_ (*p* < 0.05) while that of encapsulant proportion was not significant (*p* > 0.05) on *a*_*w*_.

#### 3.2.5. Hygroscopicity

**Figure 3:**
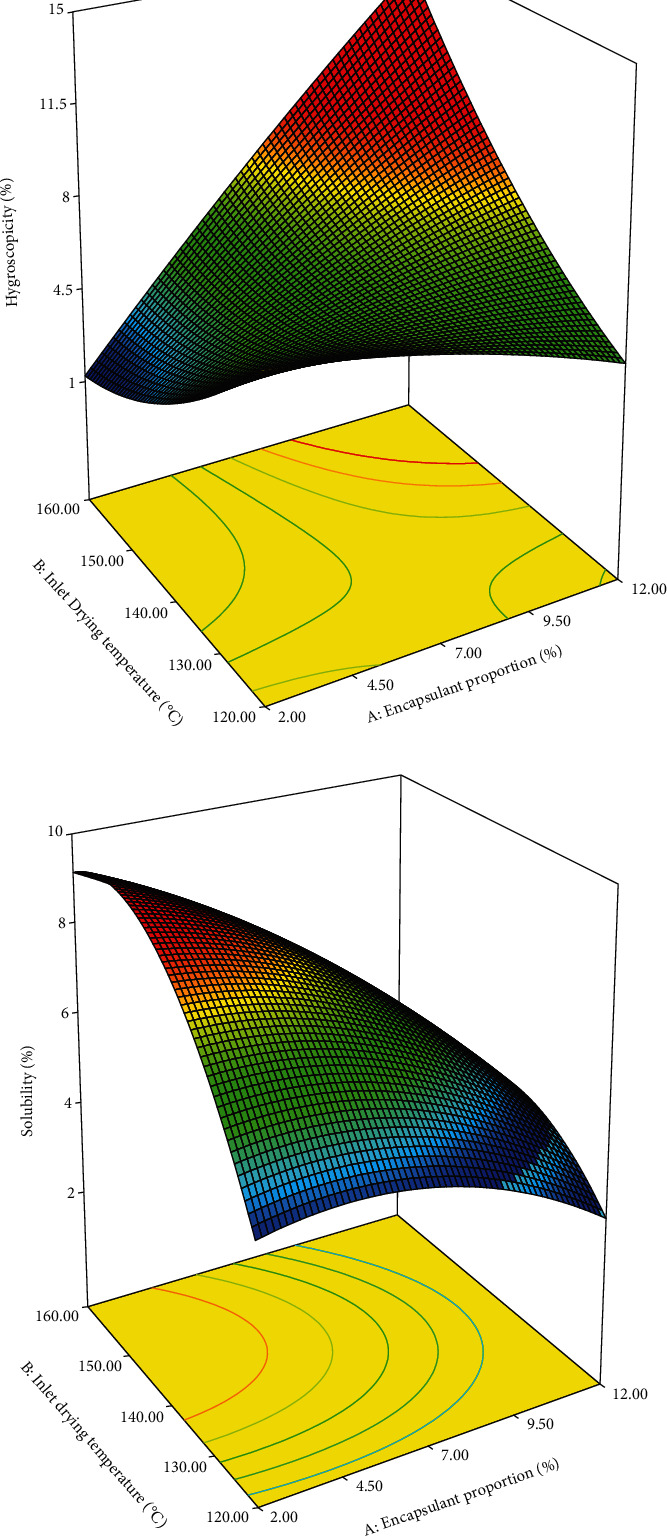
Effect of IDT and encapsulant proportion on hygroscopicity (a) and solubility (b) of PME powder.

Figure 3(a) shows the relation of IDT and encapsulant proportion with hygroscopicity of the powder. The figure shows that the powder spray dried at a high IDT and a low encapsulant proportion presented a low hygroscopicity, probably because of the effect of crust formation in the powder by the gelatinisation of the starch, which prevents the capture of water vapour [24]. Moreover, this behaviour corresponds to the high EE, total phenol content, and antioxidant capacity at high IDT and low encapsulant proportions. However, the hygroscopicity was higher at higher IDT and higher encapsulant proportion, which could be because of the increase in the porosity of granules at a higher temperature. This in turn exposes the amylose and amylopectin chains to adsorb more water. Hygroscopicity values of PME powder were lower than that of atomised powders reported by Carvalho et al. [21] and Saikia et al. [30]. This difference may be due to the type of encapsulant agent and the extract used.  Table 3 shows that the encapsulant proportion had a significant effect on hygroscopicity (*p* < 0.05), whereas that of IDT was not significant (*p* > 0.05) on hygroscopicity.

#### 3.2.6. Solubility

The solubility of atomised powder was high at high IDT and low encapsulant proportions (Figure 3(b)). High IDT helped the formation of a porous layer on the surface of the solid atomised particles, which in turn resulted in the increase of the wettability and dissolution, thus justifying the increase in solubility [24, 32]. These results indicate that the EE, total phenol content, and antioxidant capacity were high at high IDT and low encapsulant proportions. Furthermore, these results indicate that the anthocyanins, total phenol content, and other components increased the solubility of the powder. However, the powder spray dried at low IDT with high or low encapsulant proportion demonstrated low solubility (Figure 3(b)), which indicates that a porous surface structure did not form or gelatinisation did not occur completely in the OSA-modified pink oca starch, unlike in the powder spray dried at high IDT. The atomised powder of PME had a solubility range of 4.73%–9.12% (Table 2). The different solubility of powder was reported by Saikia et al. [30] and Tonon et al. [17] for açai extract powder. The difference in solubility of PME powder with other studies is possibly attributed to the starch gelatinisation and the use of OSA starch, which has hydrophobic groups.  Table 3 shows that the effects of encapsulant proportion and IDT on solubility were significant (*p* < 0.05).

### 3.3. Optimisation of Processing Conditions

Tables 3 and 4 list the analysis of variance and regression coefficients of the quadratic models for EE, antioxidant capacity, total phenol content, *a*_*w*_, hygroscopicity, and solubility of the atomised powder of PME. The quadratic models indicate that the independent variables significantly affected the EE, antioxidant capacity, *a*_*w*_, and solubility of the atomised powder (*p* < 0.05). High determination coefficients were observed for the response variables EE, antioxidant capacity, *a*_*w*_, and solubility (*R*^2^ > 0.8), whereas low determination coefficients were observed for total phenol content (0.68) and hygroscopicity (0.70). These results indicate that the response surface models can explain the variations in response variables [33]. Furthermore, the analysis of the residuals performed on the predicted data did not exceed 10% error (data not shown), indicating that the proposed quadratic models fit with the experimental data very well.

The optimal conditions of encapsulant proportion and IDT were determined using the desirability function. To obtain the optimum condition, the response variables EE, antioxidant capacity, total phenol content, and solubility were maximised, while *a*_*w*_ and hygroscopicity were minimised (Table 5). This function evaluated different encapsulant proportions and IDT to optimise the response variables. The optimum conditions obtained were 2% encapsulant proportion and 160°C IDT; using these optimum conditional values, the optimum values of the response variables were obtained (Table 5). With these optimum values of the independent variables, atomised powder with a high content of antioxidant compounds of PME could be obtained and the stability of the powder could be maintained at low *a*_*w*_ and hygroscopicity. The response variable values predicted using the optimum condition were comparable to the experimental data, thus indicating that the desirability function yielded a good prediction (Table 5).

## 4. Conclusion

As the OSA-modified pink starch showed the highest EE of monomeric anthocyanins and antioxidant capacity, it was selected as the encapsulating agent for optimising the spray drying process of PME. The derived quadratic models fit well for EE, antioxidant capacity, *a*_*w*_, and solubility of the powder. The desirability function allowed the determination of the optimum condition of the spray drying process, which was 160°C IDT and 2% encapsulant. This condition maximised EE, antioxidant capacity, total phenol content, and solubility and minimised *a*_*w*_ and hygroscopicity. The OSA-modified pink oca starch is a good option to be used as an encapsulating agent of phenolic extracts because it can produce powder with high antioxidant capacity and high EE. Furthermore, in vitro or in vivo studies are required to evaluate its behaviour in the digestive tract and the functionality of antioxidant components present in encapsulated PME.

## Figures and Tables

**Table 1:** Encapsulation efficiency, total phenol content, and antioxidant capacity of purple mashua extracts spray dried with native and OSA-modified starches.

Starch	Encapsulation efficiency (%)	Total phenol content (mg/g)	Antioxidant capacity (*μ*mol Trolox/g)
Native yellow oca	65.51 ± 0.71^c^	4.01 ± 0.08^b^	39.29 ± 0.17^c^
OSA-modified yellow oca	67.18 ± 0.64^b^	3.83 ± 0.05^c^	41.46 ± 0.14^b^
Native pink oca	65.05 ± 0.82^c^	3.73 ± 0.13^c^	41.87 ± 0.28^b^
OSA-modified pink oca	72.85 ± 0.70^a^	3.87 ± 0.07^b,c^	42.93 ± 0.21^a^
Native yellow olluco	64.41 ± 0.93^c^	4.73 ± 0.09^a^	37.76 ± 0.25^d^
OSA-modified yellow olluco	68.81 ± 1.05^b^	4.57 ± 0.10^a^	36.03 ± 0.43^e^

Data show the mean ± standard deviation. Data with different letters in the same column indicate a significant difference (*p* < 0.05). The spray drying process was performed at 140°C IDT and 4% starch.

**Table 2:** Result of the response variables of the atomised extracts.

Run	*X*_1_	*X*_2_	Encapsulation efficiency (%)	Total phenol content (mg/g)	Antioxidant capacity (*μ*mol Trolox/g)	*a*_*w*_	Hygroscopicity (%)	Solubility (%)
1	3.5	126	72.73	3.09	28.54	0.44	4.71	6.90
2	10.5	126	51.72	2.42	22.59	0.39	5.17	5.08
3	3.5	154	80.33	7.84	47.83	0.27	1.29	9.12
4	10.5	154	35.29	2.29	18.08	0.30	9.82	4.73
5	2	140	84.31	4.17	40.22	0.31	5.69	8.61
6	12	140	53.33	1.96	18.07	0.34	8.80	4.34
7	7	120	59.18	3.21	30.56	0.38	7.59	4.34
8	7	160	60.61	2.09	21.06	0.29	9.93	6.54
9	7	140	71.74	3.18	30.32	0.35	6.23	7.12
10	7	140	69.57	3.23	31.02	0.35	6.36	8.09
11	7	140	70.83	3.02	31.96	0.34	6.40	7.68
12	7	140	69.81	3.20	31.06	0.35	6.12	7.39

*X*_1_: encapsulant proportion (OSA-modified pink oca starch); *X*_2_: inlet drying temperature.

**Table 3:** Analysis of variance (significance) of CCR design for each response.

Parameter	EE	Total phenol content	Antioxidant capacity	*a*_*w*_	Hygroscopicity	Solubility
*X*_1_	1508.72^∗^	10.92^∗^	561.54^∗^	6.287 × 10^−5^	22.41^∗^	18.75^∗^
*X*_2_	5.79	1.15	0.23	0.019^∗^	2.58	3.10^∗^
*X*_11_	23.56	0.30	1.78	2.5 × 10^−4^	0.27	1.14^∗^
*X*_22_	260.61^∗^	5.63 × 10^−4^	30.84	1.0 × 10^−5^	1.95	5.66^∗^
*X*_12_	144.36^∗^	5.95	141.61^∗^	1.6 × 10^−3^	16.28	1.65^∗^
*p* value model	0.001	0.132	0.0123	0.018	0.117	0.0004
*p* value (lack of fit)	0.006	0.0003	0.0023	0.005	0.0002	0.407
*R* ^2^	0.9425	0.6896	0.8701	0.8529	0.7045	0.9605
*R* ^2^ adjusted	0.8946	0.4310	0.7619	0.7303	0.4583	0.9276
CV	6.80	35.44	14.60	7.12	26.91	6.75
PRESS	817.45	58.53	777.25	0.025	130.62	5.87

*X*_1_ and *X*_11_: linear and quadratic encapsulant proportion (OSA-modified pink oca starch); *X*_2_ and *X*_22_: linear and quadratic inlet drying temperature; *X*_12_: interaction between encapsulant proportion and inlet drying temperature; *R*^2^: coefficient of determination; CV: coefficient of variation. ^∗^Significance at *p* = 0.05.

**Table 4:** Estimates of the regression coefficients of the second-order polynomial of the CCR design for each response.

Regression coefficient	EE	Total phenol content	Antioxidant capacity	*a*_*w*_	Hygroscopicity	Solubility
*β*_0_	−659.487^∗^	−20.896	−294.38	1.076	92.036	−104.134^∗^
*β*_1_	15.433	2.904	15.208	−0.049	−5.052	1.881^∗^
*β*_2_	9.914^∗^	0.188	3.997	−4.53 × 10^−3^	−1.037	1.479^∗^
*β*_11_	−0.154	0.018	−0.043	−5.1 × 10^−4^	−0.017	−0.035^∗^
*β*_22_	−0.033^∗^	4.78 × 10^−5^	−0.011	−6.38 × 10^−6^	0.003	−0.005^∗^
*β*_12_	−0.126^∗^	−0.025	−0.1214^∗^	4.08 × 10^−4^	0.041	−0.013^∗^

1: encapsulant proportion; 2: inlet drying temperature. ^∗^Significance at *p* = 0.05.

**Table 5:** Optimum parameters of response variables obtained using the desirability function for atomised extracts.

Variable	Experimental range	Optimum value	Desirability
Independent variables			
Encapsulant proportion (%)	2–12	2	0.99
IDT (°C)	120–160	160	
Responses		Experimental value	Predicted value
Encapsulation efficiency (%)	35.29–84.31	82.69	84.31
Total phenol content (mg/g)	1.96–7.84	7.92	8.30
Antioxidant capacity (*μ*mol Trolox/g)	18.07–47.83	47.18	49.87
*a*_*w*_	0.27–0.44	0.24	0.22
Hygroscopicity (%)	1.29–9.93	1.4	1.30
Solubility (%)	4.34–9.12	9.11	9.19

## Data Availability

All the data relevant to the research can be found in the manuscript. Further information is available from the corresponding author upon request.
